# Ion exchanger in the brain: Quantitative analysis of perineuronally fixed anionic binding sites suggests diffusion barriers with ion sorting properties

**DOI:** 10.1038/srep16471

**Published:** 2015-12-01

**Authors:** Markus Morawski, Tilo Reinert, Wolfram Meyer-Klaucke, Friedrich E. Wagner, Wolfgang Tröger, Anja Reinert, Carsten Jäger, Gert Brückner, Thomas Arendt

**Affiliations:** 1 Paul Flechsig Institute for Brain Research, University of Leipzig, Liebigstrasse 19, D04103 Leipzig, Germany; 2 Physics Department, University of North Texas, 1155 Union Circle #311427, Denton, Texas 76203, USA; 3 EMBL Hamburg, Building 25A, DESY, Notkestraße 85, D22603 Hamburg, Germany; 4 Physik-Department E15, Technische Universität München, James-Franck-Straße, D85748 Garching, Germany; 5 Max-Planck-Innovation GmbH, Amalienstrasse 33, D80799 Munich, Germany

## Abstract

Perineuronal nets (PNs) are a specialized form of brain extracellular matrix, consisting of negatively charged glycosaminoglycans, glycoproteins and proteoglycans in the direct microenvironment of neurons. Still, locally immobilized charges in the tissue have not been accessible so far to direct observations and quantifications. Here, we present a new approach to visualize and quantify fixed charge-densities on brain slices using a focused proton-beam microprobe in combination with ionic metallic probes. For the first time, we can provide quantitative data on the distribution and net amount of pericellularly fixed charge-densities, which, determined at 0.4–0.5 M, is much higher than previously assumed. PNs, thus, represent an immobilized ion exchanger with ion sorting properties high enough to partition mobile ions in accord with Donnan-equilibrium. We propose that fixed charge-densities in the brain are involved in regulating ion mobility, the volume fraction of extracellular space and the viscosity of matrix components.

The extracellular matrix (ECM) together with soluble factors constitutes the microenvironment of neurons in the brain. This microenvironment sets the controlling conditions for determining the neuronal phenotype. It provides the contextual extracellular cues for tissue-specific and cell-type specific gene expression and for functional differentiation of neurons^1^,^2^,^3^,^4^. In addition, the ECM interacts with adhesive and paracrine signals from neighbouring cells, distant tissues and systemic cues. Also, it enables cellular homeostasis and electrical signalling by providing a reservoir of extracellular ions sufficient to maintain resting, synaptic and action potentials. It is, thus, the ECM physico-chemical properties that govern local concentration gradients of bioactive molecules which affect the regulation of neuronal function and viability^5^,^6^,^7^.

The ECM is composed of long-chain macromolecules many of which are linked to cell surfaces while others float in the extracellular space. Within the extracellular space, the dominant mechanism of molecular transport is diffusion, constrained by the geometry of this compartment and the physico-chemical properties of the ECM^8^,^9^. One of the major factors that determine diffusion rate and, thus local concentration within the extracellular space is the interaction with fixed negative charges on the ECM when the diffusing molecule is charged^10^.

Composition and density of the ECM vary greatly among brain regions^11^ and even in the direct microenvironment of neurons. There is a distinguished form of the ECM, the perineuronal net (PN). PNs ensheath defined subsets of neurons^12^, characterized by high firing frequency^13^ and segregate them into functional units. The PN consists of long-chain polyelectrolytes with three main components: glycosaminoglycans (e.g. hyaluronan, chondroitin sulfate and heparin sulfate), glycoproteins (e.g. tenascin), and proteoglycans (e.g. lectican family)^14^. Due to their glycosaminoglycan components, PNs may form highly negatively charged fixed structures in the direct microenvironment of neurons^15^,^16^. Through electrostatic interactions, they may, thus, significantly alter the diffusion properties and local homeostasis of physiologically relevant mobile ions.

The effects of charges provided by proteoglycans and especially their glycosaminoglycans have mainly been studied *ex vivo* in cartilage and in solutions containing extracellular matrix components^15^,^16^. Until now, locally fixed charges in the tissue have not been accessible so far to direct observations and quantifications. In particular, in the brain, the net amount and distribution of charge density as a critical determinant for local diffusion properties are still unknown^8^,^9^. Thus, assumptions on charge effects remain unsubstantiated until clear estimates about the local “ion-binding capacities” are available.

For the present study, we developed a new experimental approach to visualize and quantify fixed charge densities on brain slices with sub-micron spatial resolution using high resolution nuclear microscopy with focused proton-beam microprobes in combination with an ionic metallic probe. We combined scanning particle-induced X-ray emission spectrometry (μPIXE), with backscattering spectrometry (BS) for quantitative elemental imaging^17^. For the first time, we provide quantitative results on the pericellularly fixed negative-charge density in the extracellular space based on measured concentrations of the bound cationic probe ion.

## Results

### Elemental imaging in brain tissue

**Figure 1:**
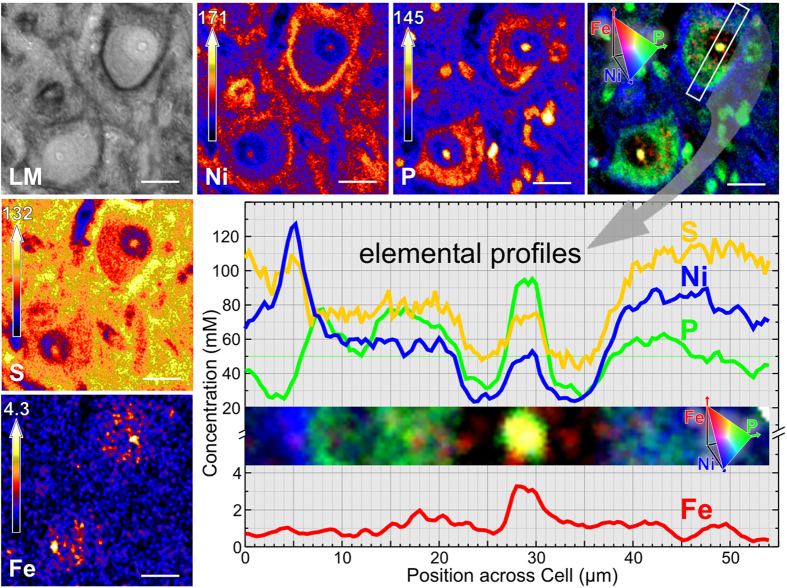
Light micrograph (LM) and quantitative PIXE elemental images of two PN-ensheathed neurons in rat brainstem (7 μm thick section, PNs visualized using WFA-binding enhanced by DAB-Ni staining (grey-black pigment)). (Ni) In the nickel map, PNs are visible due to the Ni-accumulation after immunohistochemical staining. (P) The phosphorus map mainly represents the distribution of the phosphate rich RNA and DNA, i.e. the Nissl substance in the neuronal cytoplasm and the nucleolus, but also glia cell nuclei. (S) The sulphur distribution reflects the extracellular matrix. Due to the sulfate rich chondroitin components of the PN the concentration is higher at the PN. (Fe) The iron map shows diffuse cytosolic and nuclear distribution and a prominent signal over the nucleolus. (Fe-P-Ni) In the three-element image of phosphorus (green), nickel (blue) and iron (red), iron can clearly be allocated to subcellular compartments delineated by the phosphorus image. Elemental profiles are given for the traverse through the PN ensheathed neuron. Scale bar: 20 μm, top concentration at the color scale in mM.

In order to analyze the fixed negative charge density in the neuronal microenvironment and PNs with an iron probe by μPIXE, the PN must be identified. Therefore, we applied an approach recently developed in our lab^18^ based on the application of antibodies tagged with an ultra-pure metallic label (e.g. Ni, Co, Cd, Ag, or Au), which combines immunohistochemistry and elemental imaging using μPIXE. Typical quantitative elemental images of brain sections obtained with this approach are shown in Fig. 1. PNs are identified in the nickel image. It shows the nickel accumulation due to the selective WFA-DAB-Ni-binding. The elemental profiles shown in Fig. 1 and control measurements (Supplementary Figure S1) verify that the Ni-enhancement of the histochemical staining does not affect the elemental imaging or the quantitative analysis of iron or other elements. The iron and nickel distributions are uncorrelated. Thus, an alteration of the iron concentration by the nickel-DAB-staining can be excluded.

The phosphorus distribution (Fig. 1-P) sharply delineates cytoplasm, nucleus, and nucleolus. Phosphorus is higher concentrated in the cytoplasm and in the nucleolus, while the nucleus shows a lower concentration. In general the phosphorus image very much resembles microphotographs of Nissl stained sections because of the staining of nucleic acids (RNA and DNA) which are rich in phosphorus due to their phosphate backbones. The phosphorus rich spots outside the neurons are nuclei of glia cells. The sulphur concentration is higher in the extracellular matrix than in the cytoplasm. PNs typically show a prominent sulphur-signal, related to chondroitin sulfate proteoglycans, the sulphated molecular components of PNs. The merged distribution of phosphorus, nickel and iron represented in an RGB false colour three-element image sets the elements in spatial context to each other. As an example for iron analysis, the concentrations within the two neurons (upper right/lower left) are given. The total cellular iron concentrations are 0.85 mM and 0.75 mM, with cytoplasmic iron (0.80 mM/0.69 mM), iron in the nucleus (1.0 mM/0.96 mM), and in the nucleolus (3.0 mM/3.1 mM). The minimum detection limit (MDL) for iron analysis in the cellular area is 18 μM. The MDLs for other elements of interest are given in Table 1.

### Mapping of anionic binding sites using Fe^3+^-ions as probe

**Figure 2:**
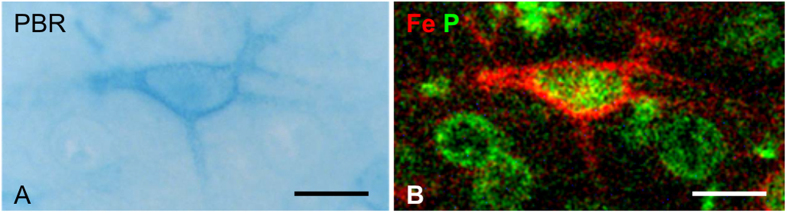
Brain section loaded with 5 mM FeCl_3_ and stained with Prussian blue reaction (PBR). (**A**) Light microscopy image of a neuron with a PN on which iron is accumulated. (**B**) Corresponding quantitative PIXE element image of phosphorus (green) and iron (red). The PN shows prominent iron accumulation (red). Cellular somata are identified by the phosphorus signal (green). Scale bar: 20 μm.

**Figure 3:**
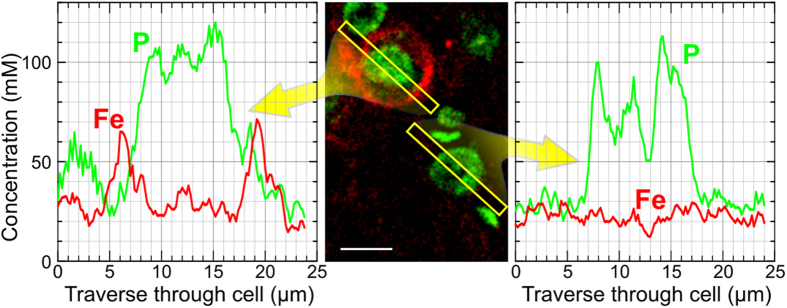
Elemental profiles of Fe and P across the somata of two neurons one with PN (upper cell) and one without PN (lower cell) extracted from PIXE element imaging data (tissue loaded with 1.68 mM FeCl_3_). The PN (previously identified within the Ni-map, not shown) localized on the outer surface of the soma shows a local increase in iron concentration several fold above the surrounding extracellular matrix and neuropil. Scale bar: 10 μm.

Anionic “binding sites” fixed to PNs were mapped and analyzed using quantitative elemental imaging of the iron distribution after adding Fe^3+^-ions as cationic probes. As a visual control but not for the analysis, Fe^3+^-ions bound to PNs were histochemically stained by the Prussian blue reaction, resulting in a delicate intense blue staining of PNs against a light blue background (Fig. 2A). The corresponding iron distribution in the element image clearly delineates PNs and matches exactly the pattern of the Prussian blue stained iron bound to PNs (Fig. 2B). With the superimposed phosphorus distribution that shows cellular somata, neurons ensheathed by PNs can be distinguished from neurons devoid of PNs. For the analysis of negative charge densities, Prussian blue staining was omitted. Taking a traverse across the two types of neurons their different ability to bind iron in the perineuronal space is demonstrated (Fig. 3).

The quantitative information on the concentrations of iron combined with that of sulfur in the perineuronal net allowed us to determine the number of iron atoms per sulphur atom, which gives an estimate of the relative amount of iron ions bound to the chondroitin sulphate disaccharide units. For the maximum iron load of 12.5 mM, the relative amount of iron atoms per sulfur atoms was with 3.8 ± 0.4 highest in the PN of the subiculum and with 1.3 ± 0.1 lowest for the PN in the red nucleus.

**Figure 4:**
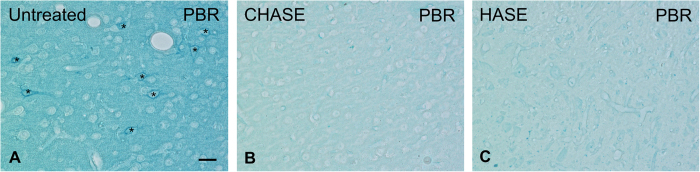
Prussian blue reaction (PBR) on brain slices loaded with 12.5 mM FeCl_3_. (**A**) untreated; (**B**; CHASE) pre-treated with chondroitinase ABC; and (**C**; HASE) pre-treated hyaluronidase. PNs are distinct (asterisks) in the untreated section (**A**), while treatment with either chondroitinase (**B**) or hyaluronidase (**C**) prior to Fe^3+^-loading abolished PN labeling. Scale bar: 20 μm, applies to all.

When sections are treated with either chondroitinase or hyaluronidase, which are known to largely remove charged glycosaminoglycan side chains of the PN, subsequent loading with Fe^3+^-ions does not lead to perineuronal labeling. Thus, intact PNs are necessary for perineuronal iron-binding (Fig. 4).

### Kinetics of Fe^3+^-binding to perineuronal nets (PNs)

**Figure 5:**
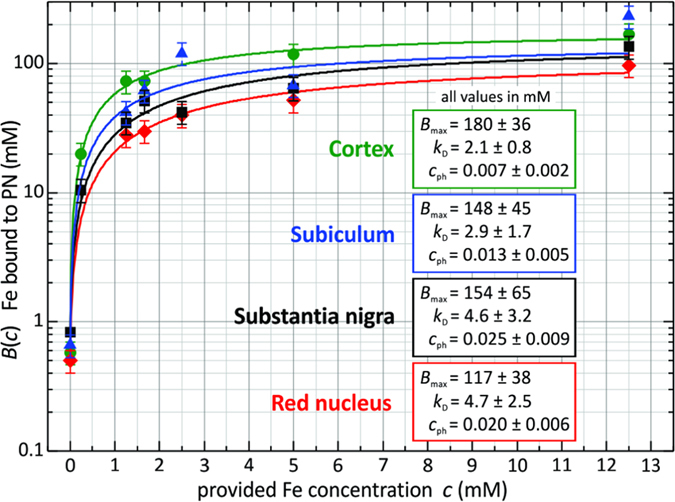
Concentration of iron bound to perineuronal nets in rat cortex, subiculum, substantia nigra and red nucleus as a function of the iron concentration applied. The relation can be described by a Langmuir adsorption equation. The stated errors are 20% of the average values from three PNs per applied iron concentration.

The dynamic binding characteristic of Fe^3+^ to the perineuronal anionic binding sites was determined using quantitative elemental imaging of tissue sections loaded with increasing concentrations of the Fe^3+^-probe. PNs accumulate more Fe than any other ECM component, which amounts to two- to three times that of ECM structures outside PNs. The affinity of the Fe^3+^-probe binding to PNs was extracted from concentration values of Fe accumulated at PNs. The relationship between the concentration of the bound Fe-probe to the applied load can be described by a Langmuir adsorption equation (Fig. 5). The relationship is given by *B*(*c*) = *B*_max_ (*c* + *c*_ph_)/(*K*_D_ + *c + c*_ph_), with *B* being the concentration of PN bound iron and *c* is the iron load applied. *c*_ph_ accounts for the low physiological free Fe concentration. The parameter *K*_D_ represents the load concentration at which the concentration of bound iron is half of its maximum value. *K*_D_ is the inverse of the affinity or Langmuir constant. The *K*_D_ and saturation values were extracted for the four rat brain regions cerebral cortex, subiculum, substantia nigra and red nucleus (Fig. 5).

### Binding characteristics of Fe^3+^ probe bound to perineuronal nets (PNs)

To conclude on charge density based on information on the molecular binding characteristics of Fe bound to PNs, we validated the binding of Fe-ions in trivalent oxidation state by extended X-ray absorption fine structure (EXAFS) analysis and by Mössbauer spectroscopy (see Supplementary Information). The analysis of the position of the X-ray absorption edge and variations in the X-ray absorption by the iron atoms in the sample yields information on the charge state, the average coordination numbers, and distances to neighbouring atoms. The results show trivalent iron in the iron loaded cortex and red nucleus samples as well as in the iron solution that was applied to the brain samples. Further analysis of the EXAFS oscillations (see Supplementary Results S2) resulted in an empirical structure indicating an Fe-O-cluster with coordination number five and Fe-O-distance of about 1.98 Å. The coordination number eight with carbon atoms and an Fe-C-distance of about 3.7 Å indicate a local organic environment. Thus, the iron is associated with the PN. A potential Fe-Cl-cluster could be excluded because the PIXE maps showed no elevated Cl concentrations at the iron loaded PNs.

## Discussion

It is the structure and composition of the neuronal microenvironment in form of the extracellular space that determines how molecules migrate through the brain. Recent estimates indicate that the extracellular space occupies at least 15% to 20% of brain tissue, with a large regional variation of its actual width^19^. Parameters of diffusion through the extracellular space vary in different parts of the CNS. There are several potential mechanisms how the ECM composition can modify the diffusion parameters of the extracellular space^9^. These relate to geometry, viscosity and specific receptor binding. For charged molecules, diffusion is in particular influenced by fixed negative charges of the ECM, which have highest densities at PNs. One way is the charge discrimination by the ECM through its anionic binding sites mediating rapid equilibrium binding of cations. Accordingly, molecules briefly leave the diffusion process and accumulate locally, representing an obstacle for free diffusion. A charge density high enough that fixed anionic charges tend to partition mobile ions in accord with Donnan equilibrium, however, had previously not been considered for brain tissue.

Here we directly visualize and quantify perineuronally fixed anionic binding sites in brain tissue using a metallic ionic probe in combination with high resolution nuclear microscopy with focused proton-beam microprobes that offers one of the best sensitivity and spatial resolution for *in situ* microanalysis of trace metals currently available^20^. PIXE is a multielemental technique with particularly high sensitivity covering all biologically relevant elements when combined with proton backscattering spectrometry. It thus allows obtaining fully quantitative images of elemental distributions, making it particularly attractive for analyzing biological samples at high spatial resolution^21^.

Anionic charge density in the microenvironment of PN-ensheathed neurons is high enough to mediate the binding of trivalent Fe with maximal binding-capacity of 117 to 180 mM, corresponding to a local charge density of around 0.4 to 0.5 M [0.351 to 0.540 M]. This indicates that the locally circumscribed charge densities in the brain is much higher than previously assumed and is well in the range or even above that for cartilage proteoglycan, where it has been estimated at 0.1 M [0.08–0.16 M]^22^.

A supposed function of PNs in maintaining the extracellular space but hindering free diffusion through reversible ionic binding is in agreement with evidence that comes from a series of experiments from the Syková lab and Nicholson lab^8^,^9^. While overall, diffusion properties in the neocortex are predominantly isotropic, the somatosensory and auditory cortex, two areas highly enriched in PN, show anisotropic diffusion properties. This link between anisotropy in diffusion and PNs is further supported by the loss of anisotropic diffusion properties in the aging hippocampus which goes along with a decrease of chondroitin sulfate proteoglycans, which are predominant components of PNs^23^. Accordingly, injections of the enzyme chondroitinase cleaving the negatively charged components of PNs^8^ or knock out of the hyaluronan-binding link protein, Bral1 that complexes with negatively charged ECM proteins^24^ facilitate diffusion. On the contrary, diffusion is slowed down by the addition of hyaluronic acid, the major anionic glycosaminoglycan components of PNs, indicating a potential retardation of diffusion through PNs. A recent study on knockout mice for tenascin-R, a major component of PNs, shows that the extracellular space volume fraction was reduced by 22–26%, while the tortuosity, as a measure for diffusion obstacles, was also reduced^25^. This supports a role for PNs in maintaining the extracellular space but serving as diffusion barrier at the same time.

The concept of hindering free diffusion through electrostatic interactions with fixed charges in the extracellular space is in agreement with studies that have shown that strong electrostatic interaction retards the diffusion of cations, at least at low ionic strength^22^,^26^. The fact, moreover, that glycosaminoglycans and proteoglycans can bind significant amounts of divalent cations such as Ca^2+^ has been shown by a number of investigators^27^,^28^,^29^ and charge-based interactions between transported molecules and the microenvironment have well been documented in cartilage^22^,^30^ and in the glycocalyx surface layer on capillary endothelial cells^31^.

In the present study, we have determined the partition coefficient [affinity-constant (*K*_D_)] for binding of trivalent Fe to PNs at 2.1 mM to 4.7 mM. This “low affinity” supports the concept of electrostatic interaction^8^,^32^.

This indicates that through ionic interactions, polyanionic components of PNs are able to reversibly accumulate cationic molecules at physiological concentrations, potentially contributing to local molecular gradients of physiologically relevant ions such as Ca^2+^, K^+^, or Na^+ ^16.

PNs may thus represent a physiological adaption to special requirements in the microenviroment of a subset of neurons. Neurons ensheathed by PNs are characterized by a unique set of electrophysiological properties, comprising low input resistance, high resting membrane potential, short action potentials and high refractory periods, and high firing frequency, suggesting their classification as fast–spiking neurons^13^. Accordingly, they are equipped with Kv3.1b, a subunit of voltage-gated potassium-channels supposed to be responsible for the generation of high firing rates^33^. These electrophysiological properties may require an extracellular reservoir for calcium, potassium and sodium that can quickly be mobilized after being trapped by the anionic binding sites of PNs.

It is well established, moreover, that neuronal activity causes a transient decrease in the volume fraction of the extracellular space, and the main cause for this reduction may be the swelling of astrocytes, possibly in response to the stimulus-induced rise in K^+^. High neuronal firing rates of fast-spiking neurons may thus require an extracellular reserve space as volume buffer to quickly compensate for swelling of astrocytes, a requirement that can be met by the hydration of the hyaluronan component of PNs. In addition, it has been suggested that changes in the concentration of ions in the microenvironment of polyelectrolytes may lead to an extension or contraction of the polyelectrolyte^34^. The largest effect on the viscosity increment of connective tissue polysaccharides in the presence of monovalent ion occurs in the range of ionic strength between 10^−4^ and 10^−2^ M^34^ which is well in the range of ionic strength we determined as partition coefficient [affinity-constant (*K*_D_)] for PNs.

One of the most conspicuous feature of PNs is their uneven distribution throughout the brain being clustered in certain areas while only scarcely present in others^12^. One consequence of this inhomogeneity of charge densities, fixed to PNs, is that they introduce a Donnan-type of distribution between their compartments and neighbouring compartments of low polyionic strength. In terms of ion distribution, the counter-cation concentration will be higher in the PN-compartment and that of the co-anion lower. Such distributions have been observed in cartilage^30^,^35^ and are in agreement with studies of Donnan distributions performed in model systems containing proteoglycans^36^.

This Donnan-type of exclusion, resulting in ‘ionic sorting’, associated with a relative barrier for small anions might have implications, highly relevant to cellular homeostasis both under physiological and pathological conditions. The perineuronally located Donnan-equilibrium will provide an inward directed force on Cl^−^-ions and an outward directed force on K^+^, thereby generating ion concentrations gradients across the membrane. This charge separation, most likely, contributes to the high resting membrane potential of neurons ensheathed by PNs. Moreover, providing a mechanism for generating K^+^ and Cl^−^ gradients, independently of the energy charge of the neuron, it will contribute to the ability of these neurons to generate high frequency action potentials^33^.

Scott^37^ proposed a survival value of an “anionic shield”. He suggests that the highly reactive hydrated electron, would be excluded from the domain of the charged polysaccharides by a Donnan mechanism.

The “anionic shield” may thus represent a mechanism protecting cells against deleterious effects of “toxic” anionic species generated in their environment by metabolic or oxidative stress, as for example, [glutamate]^−^ or [OH]^−^. This concept is supported by recent observations on neuroprotective properties of PN-associated chondroitin sulfate proteoglycans^5^,^6^,^38^,^39^,^40^,^41^. As we showed recently^5^, the lack of specific components of the PN (aggrecan, link protein or tenascin-R) or the removal of the high negative charge of PNs provided by the high degree of sulphation of the CS glycosaminoglycan chains^40^,^42^, are essential factors for the neuroprotective function of PNs^43^.

While the physiological function of PNs is not entirely understood, they are generally considered to play a role in restricting synaptic plasticity^44^. This process involves interaction with bioactive molecules such as Semaphorin3A^45^,^46^,^47^ and Otx2^48^ which both binds to PNs, depending on the sulfation pattern of their chondroitin sulfate proteoglycan components^48^. Our technique will open up a new approach to analyze whether ion sorting properties of PNs may be involved in the regulation of synaptic plasticity, which could be relevant for long-term memory consolidation^49^.

## Material and Methods

The animals used in this study were treated in agreement with the German law on the use of laboratory animals and followed the ethical guidelines of the Laboratory Animal Care and Use Committee at the University of Leipzig. Adult rats were anesthetized with pentobarbital (50 mg/kg) and transcardially perfused with saline containing heparin, followed by fixative (4%) paraformaldehyde in a 0.1 M phosphate buffer at pH 7.4. Brains were removed from the skull and dissected into 5 mm thick blocks that were postfixed overnight at 4 °C, embedded in paraffin, and cut into 6 μm thick sections. After deparaffinization, sections were incubated in iron hydroxide (boiled FeCl_3_) for 30 min under acidic conditions at a pH = 2.3 (5% acetic acid) to provide free Fe^3+^ ions. A series of increasing ion loads was prepared with the following concentrations: 0.25 mM, 1.26 mM, 1.68 mM, 2.5 mM, 5.0 mM and 12.6 mM. Bound Fe was visualized by Prussian blue reaction for light microscopy only.

For histochemical detection of PNs, sections were stained with the N-acetylgalactosamine-specific, biotinylated Wisteria floribunda agglutinin (WFA) (BioWFA; Sigma, Germany; 1:250). To detect lectin staining of PNs both at light microscopy and μPIXE-imaging, labeling was processed by nickel enhanced staining of 3,3’diaminobenzidine (DAB) (purity grade of nickel 99.999%; Sigma)^18^. Brain slices were embedded in mounting medium (DePeX, Merck) and mounted onto specific sample holders.

To prove that the preparation and staining procedure does not affect the endogenous Fe-concentration of the slice, a pair of consecutive DePeX-embedded sections, one stained, the other unstained, were prepared and measured for comparison. Additionally, 10 μm thick brain slices from a fresh, unfixed rat brain were cut on cryomicrotome, mounted on glass slides, embedded in DePeX after air-drying and mounted on sample holder to measure the tissue iron concentration. In both controls, in the paraffin embedded sections as well as in cryosections, the Fe-concentration in the total scan area was equal to that of the stained samples. For PIXE and EXAFS, slices remained unstained, were embedded in DePeX and mounted onto sample holders.

### Nuclear microscopy for quantitative elemental imaging

Scanning particle induced X-ray emission (μPIXE) spectrometry and backscattering spectrometry (BS) are mega-electronvolt (MeV) ion beam analytical techniques that provide a standardless determination of absolute concentrations of elements throughout the periodic table. In scanning mode quantitative elemental imaging can be performed, which is usually referred to nuclear microscopy^50^ (Ryan NIMB 2011).

PIXE is based on the capability of MeV-ions, in this study protons, to ionize the sample atoms mainly in the K- and L-shell. The subsequent emission of element specific, i.e. characteristic X-rays yields information on the concentration of a particular element in the sample via the intensities of its characteristic X-ray lines (peaks in the spectrum).

Backscattering spectrometry (BS) relies on measuring the energy of protons backscattered by atomic nuclei, which allows for the determination of the organic composition (C, N, and O, that of H indirectly) of the sample, and thus permits the simultaneous detection of both low and high atomic number (*Z*) elements when combined with PIXE^51^.

Nuclear microscopy was carried out at the Leipzig microprobe laboratory LIPSION using a 2.25 MeV proton beam focused to 0.5 μm and 1 μm spot sizes at beam currents of about 120 pA and 1000 pA, respectively. PIXE was used for simultaneous multi-elemental analysis of elements with atomic numbers greater than that of sodium. Simultaneously, information on the matrix composition was obtained by BS to account for matrix effects in PIXE quantitative analysis. The μPIXE analysis software, GeoPIXE, uses a fundamental parameter approach and dynamic analysis for quantitative elemental imaging and provides various tools for the extraction of quantitative results from the images. The regions of interest for the analysis of the elemental concentrations at PNs were chosen to include areas where the Fe concentrations exceeded 40% of the maximum.

### Extended X-ray absorption fine structure (EXAFS)

EXAFS experiments on cortex and red nucleus samples loaded with 2.5 mM iron hydroxide were performed at the synchrotron radiation beam line of the EMBL (European Molecular Biological Laboratories)-Outstation at the storage ring DESY (Deutsches Elektronen Synchrotron) in Hamburg to confirm the Fe(III) charge state and gain information on the molecular environment of the Fe^3+^-probe bound to PNs. The spectrometer is specifically suited for measurements on biological and other dilute systems. The basic optical system consists of a Si-double monochromator followed by a horizontally focusing mirror. All optical elements are located inside a vacuum system, which is separated from the storage ring by two 0.4 mm thick Be windows. Bragg reflections from a static Si crystal mounted at the very end of the spectrometer are recorded simultaneously with the spectra and allow calibrating absolutely the energy axis. After passing through a set of horizontal and vertical slits and a 5 μm thick Al-foil (photoemission monitor of primary intensity) the beam is diffracted from a temperature controlled double crystal monochromator (optional Si(111) or Si(220)) through another set of vertical slits onto a toroidal mirror, which produces a slightly demagnified (M = 0.8) source image at 34 m. Optional the mirror can be moved down to allow the monochromatic beam to be used directly. A slit system (vertical and horizontal), sealed ionization chambers, a modified 2-stage Displex cryostat, a fluorescence detector and the energy calibrating system are mounted on an optical bench inside the experimental hutch. Samples are located in a He exchange gas volume inside the cryostat. Control of the experiment and data acquisition is based on CAMAC modules in combination with an IBM compatible PC.

### Ethics Statement

The use of the animals for the experiments and the methods were carried out in accordance with approved guidelines and had been approved by the local authorities Saxony (Landesdirektion Sachsen, Leipzig, Germany) based on the recommendation of the advisory Ethics Commission (T44/04, T51/08; Landesdirektion Sachsen, Leipzig, Germany). The treatment of the animals was carried out in accordance with the then in force European Council Directive 86/609/EEC and with the Directive 2003/65/EC (on the amendment of the former) and in accordance with the German Animal Welfare Act (TierSchG).

## Additional Information

**How to cite this article**: Morawski, M. *et al.* Ion exchanger in the brain: Quantitative analysis of perineuronally fixed anionic binding sites suggests diffusion barriers with ion sorting properties. *Sci. Rep.* **5**, 16471; doi: 10.1038/srep16471 (2015).

## Supplementary Material

Supplementary Information

## Figures and Tables

**Table 1:** Minimum detection limit (MDL) in units of μM derived from Fig. 1.

Element	P	S	Cl	K	Ca	Mn	Fe	Ni	Cu	Zn	Br
for total area	64	52	41	28	21	6.0	5.5	15	20	17	82
for 1000 μm^2^	204	163	129	89	65	19	18	48	62	53	259
for 1 μm^2^	6400	5100	4100	2800	2100	600	550	1500	1900	1700	8200

The MDL depends on the size of the region of interest. The neurons in Fig. 1 are about 1000 μm^2^ in size.
