# Supplementary material for: Ion exchanger in the brain: Quantitative analysis of perineuronally fixed anionic binding sites suggests diffusion barriers with ion sorting properties
Source: Sci Rep. 2015 Dec 1;5:16471. doi: 10.1038/srep16471 (PMC4664884; doi:10.1038/srep16471)
Supplement: Supplementary Information [file srep16471-s1.doc]

**Supplementary information to the manuscript:**

**Ion exchanger in the brain: Quantitative analysis of perineuronally fixed anionic binding sites suggests diffusion barriers with ion sorting properties**

**Short title: Ion exchanger in the brain**

**Markus Morawski ^a §^ , Tilo Reinert ^b §^ , Wolfram Meyer-Klaucke ^c^, Friedrich E. Wagner ^d^, Wolfgang Tröger ^e^, Anja Reinert ^a^, Carsten Jäger ^a^**, **Gert Brückner ^a^ and Thomas Arendt**

**a #**

**^a^** Paul Flechsig Institute for Brain Research, University of Leipzig, Liebigstrasse 19, D04103 Leipzig, Germany, **^b^** Physics Department, University of North Texas, 1155 Union Circle #311427, Denton, Texas 76203, USA, **^c^** EMBL Hamburg, Building 25A, DESY, Notkestraße 85, D22603 Hamburg, Germany, **^d^** Physik-Department E15, Technische Universität München, James-Franck-Straße, D85748 Garching, Germany, and **^e^** Institute for Experimental Physics II, University of Leipzig, Linnéstrasse 5, D04103 Leipzig, Germany

**This file contains:**

Supplementary figures S1 and S2, material and methods as well as all results (S3 and table ST1) of the Mössbauerspectroscopy.

**S1 Combining PIXE trace element analysis with immunohistochemistry**


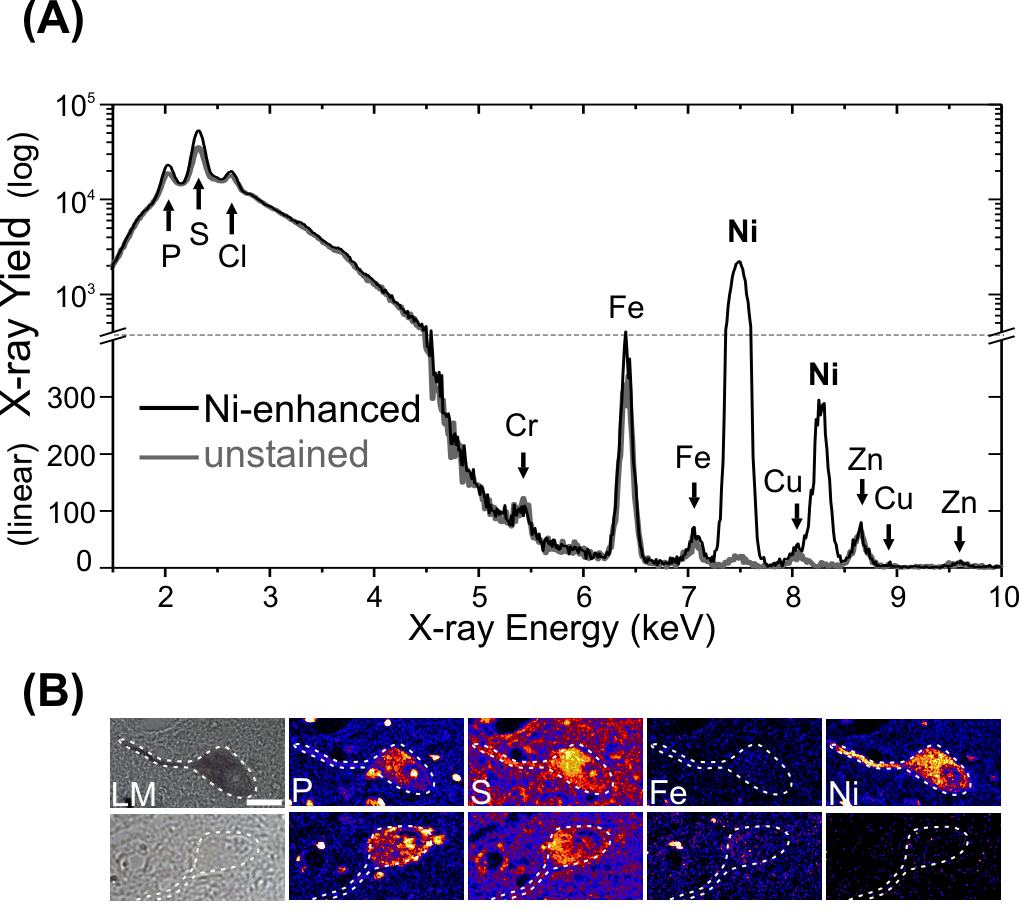


In the present study, we used nickel ammonium sulphate with a purity of 99.999% for nickel enhanced DAB staining which is a sufficient purity to avoid artefactual contamination influencing trace element analysis. Supplementary figure S1(A) shows two PIXE-spectra comparing the elemental content of an unstained brain section of the rat substantia nigra pars compacta with its consecutive section labelled with a Ni-enhanced antibody (anti-tyrosine hydroxylase) reaction. There is no significant influence by the immunocytochemical reaction onto trace element concentration except the intentional Ni-enhancement. These results show that for spatially resolved quantitative analysis of elements (e.g. P, S, Fe, Ni) using μPIXE the immunohistochemistry enhanced with a specific metal such as Ni, is a suitable and versatile tool to assist in the localisation and differentiation of particular structures allowing for a combination of light microscopy and μPIXE mapping S1(B).
Scale bar in S1(B): 20 μm

**S2 Supplementary results (EXAFS measurements)**

**S2.1 EXAFS analysis of Cortex with 2.5 mM FeCl_3_**

| **Cortex** | | | |
| --- | --- | --- | --- |
| **Path** | **Coordination Number** | **Interatomic**  **Distance (Å)** | **Disorder**  **(10^-3^ Å^2^)** |
| Fe-O | 5 | 1.983(3) | 19.9(6) |
| Fe-Fe_1_ | 2 | 3.000(7) | 24.58(2) |
| Fe-Fe_2_ | 4 | 3.432(6) | 20(1) |
| Fe-C | 8 | 3.69(1) | 18(4) |

**S2.2 EXAFS analysis of Red Nucleus with 2.5 mM FeCl_3_**

| **Red Nucleus** | | | |
| --- | --- | --- | --- |
| **Path** | **Coordination Number** | **Interatomic**  **Distance** (Å) | **Disorder**  (10^-3^ Å^2^) |
| Fe-O | 5 | 1.982(3) | 17.86(5) |
| Fe-Fe_1_ | 2 | 2.996(6) | 22.4(1) |
| Fe-Fe_2_ | 4 | 3.441(7) | 21(1) |
| Fe-C | 8 | 3.71(1) | 21(4) |

**S2.3 EXAFS analysis of FeCl_3_ aqueous solution**

| **Aqueous FeCl_3_ Solution** | | | |
| --- | --- | --- | --- |
| **Path** | **Coordination Number** | **Interatomic**  **Distance** (Å) | **Disorder**  (10^-3^ Å^2^) |
| Fe-O | 5 | 1.983(3) | 19.9(6) |
| Fe-Cl | 2 | 3.111(6) | 10(1) |

**Material and Method- Mössbauer spectroscopy**

**Mössbauer spectroscopy.** ^57^Fe Mössbauer spectra were measured for a cortex and a red nucleus specimen with 5 mM iron hydroxide load. The measurements were taken at RT, 160 K (cortex), 170 K (red nucleus), and 4.2 K (both). The absorbers had a thickness of 123 mg/cm2 for the cortex and of 142 mg/cm2 for the red nucleus sample. The source of 57Co in rhodium was always kept at the temperature of the absorber. All isomer shifts will be given as measured, i.e., with respect to the source having the same temperature as the absorber. The spectra were fitted with appropriate superpositions of Lorentzian lines allowing for a Gaussian distribution of the magnetic hyperfine field or the electric quadrupole splitting, respectively.

**Results-Mössbauer spectroscopy**

Further information on the chemical state of the PN-bound Fe-cations and characterization of the local environment of the bound iron probe were obtained by Mössbauer spectroscopy. The Mössbauer spectra of the cortex at RT and 160 K and the red nucleus at RT and 170 K both exhibit quadrupole doublets (red signals in Fig. S3 A, B, D, E) with median isomer shifts of 0.20(2) mm/s and quadrupole splittings with median values from 0.66(2) mm/s to 0.68(2) mm/s. The variances are near 0.3 mm/s. At 4.2 K the doublets have disappeared and six-line magnetic hyperfine patterns have emerged (red signals in Fig. S3 C and F). This transition, from a doublet to a sextet at lower temperatures, is characteristic for superparamagnetism suggesting the existence of nanoparticles with magnetic ordering. The magnetic hyperfine patterns were fitted allowing for a Gaussian distribution of the magnetic hyperfine field. The median field values were found to be 48.9(1) T and 49.0(1) T for the cortex and the red nucleus, respectively, with variances of 2.2(1) T and 1.9(1) T. The isomer shifts of 0.24(1) mm/s of the magnetically split patterns are typical for trivalent Fe. The quadrupole splittings in the magnetic patterns are close to zero. The existence of iron nanoparticles in the samples is a consequence of the drying process that was necessary for the analysis. The precipitates have formed when the solvent dried out.

The quadrupole doublets visible in the 4.2 K spectra of the cortex ( dark grey doublet in Fig. S3 C) and the red nucleus (dark grey doublet in Fig. S3 F) have isomer shifts of ‑0.16(1) mm/s and ‑0.12(1) mm/s with quadrupole splittings of 0.53(2) mm/s and 0.57(2) mm/s, respectively. It is attributed to iron impurities in the beryllium window of the proportional counter and thus have no relation to the properties of the iron in the brain samples. When this component is properly taken into account in the fitting of the spectra taken at 160 K and RT, the quadrupole doublets of the iron in the brain specimens all have median isomer shifts of 0.20(2) mm/s, while the quadrupole splittings have median values of 0.66(2) mm/s and variances of near 0.3 mm/s. All Mössbauer spectra show no trace of divalent iron, which would be expected to give rise to a quadrupole doublet with a shift of about 1 mm/s and a splitting of more than 2 mm/s (for summary see Table T1). However, the red nucleus sample shows an additional six-line magnetic hyperfine pattern at all temperatures (light grey sextet in Fig. S3 D, E, F). This signal with a magnetic hyperfine field of 34.0 T at 4.2 K and 33.0 T at RT originates from another form of iron, metallic α-Fe. Since our EXAFS analysis contrarily did not reveal any signatures of α-Fe we believe the sample of the red nucleus prepared for Mössbauer was contaminated with metallic iron during the preparation of the brain tissue with the metallic surgical instruments.

The Mössbauer spectroscopy measurements complement the EXAFS results and provide clear evidence that Fe was bound to PNs in its trivalent oxidation state.


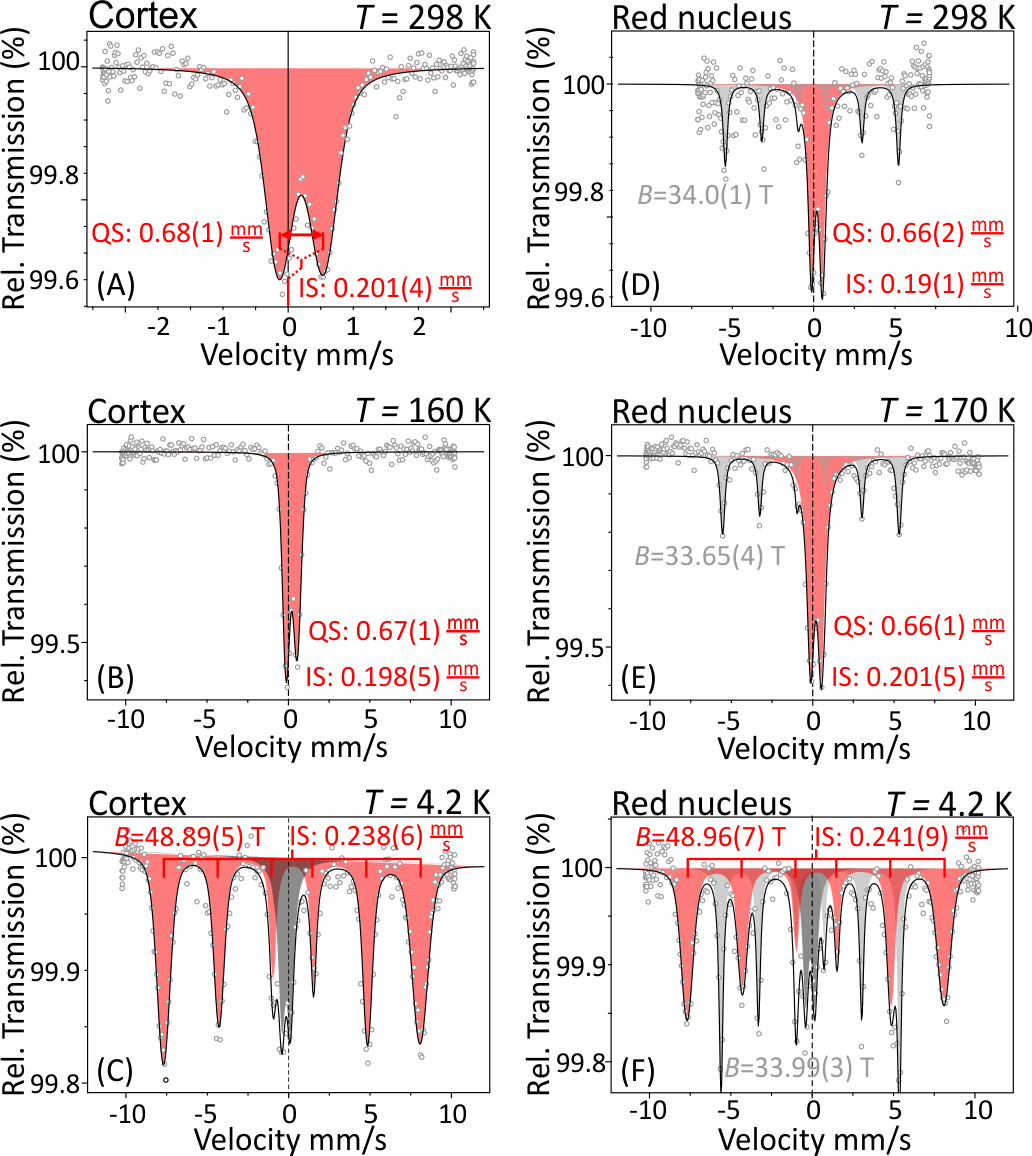


**Figure S3**

Mössbauer spectra of (A, B, C) rat cortex and (D, E, F) red nucleus loaded with 2.5 mM FeCl_3_ taken at RT (top), 160 K (middle) and at 4.2 K (bottom).

**Supplementary Table T1:**

Summary of Mössbauer data from Cortex and Red Nucleus samples.

| **Sample** | **Temperature** | **Lines** | **IS (mm/s)** | **QS (mm/s)** | ***B*_hf_  (T)** |
| --- | --- | --- | --- | --- | --- |
| Cortex | 298 K | Doublet | 0.200(4) | 0.683(7) |  |
|  | 160 K | Doublet | 0.197(4) | 0.674(7) |  |
|  | 4.2 K | Sextet | 0.24(1) |  | 48.88(5) |
|  |  | Doublet ^a)^ | – 0.16(1) | 0.53(2) |  |
| Red Nucleus | 298 K | Doublet | 0.19(1) | 0.66(2) |  |
|  |  | Sextet ^b)^ | – 0.11(1) |  | 33.00(8) |
|  | 170 K | Doublet | 0.200(5) | 0.663(8) |  |
|  |  | Sextet ^b)^ | –0.109(6) |  | 33.65(4) |
|  | 4.2 K | Sextet | 0.242(9) |  | 48.96(7) |
|  |  | Doublet ^a)^ | – 0.12(1) | 0.57(2) |  |
|  |  | Sextet ^b)^ | – 0.125(5) |  | 33.99(3) |
